# Direct nanoscale observations of the coupled dissolution of calcite and dolomite and the precipitation of gypsum

**DOI:** 10.3762/bjnano.5.138

**Published:** 2014-08-11

**Authors:** Francesco Giancarlo Offeddu, Jordi Cama, Josep Maria Soler, Christine V Putnis

**Affiliations:** 1 Institute of Environmental Assessment and Water Research (IDAEA), CSIC, Jordi Girona 18-26, 08034 Barcelona, Catalonia, Spain; 2 Institut für Mineralogie, University of Münster, Corrensstrasse 24 D-48149, Münster, Germany

**Keywords:** atomic force microscopy (AFM), calcite, dissolution–precipitation, dolomite, gypsum

## Abstract

In-situ atomic force microscopy (AFM) experiments were performed to study the overall process of dissolution of common carbonate minerals (calcite and dolomite) and precipitation of gypsum in Na_2_SO_4_ and CaSO_4_ solutions with pH values ranging from 2 to 6 at room temperature (23 ± 1 °C). The dissolution of the carbonate minerals took place at the (104) cleavage surfaces in sulfate-rich solutions undersaturated with respect to gypsum, by the formation of characteristic rhombohedral-shaped etch pits. Rounding of the etch pit corners was observed as solutions approached close-to-equilibrium conditions with respect to calcite. The calculated dissolution rates of calcite at pH 4.8 and 5.6 agreed with the values reported in the literature. When using solutions previously equilibrated with respect to gypsum, gypsum precipitation coupled with calcite dissolution showed short gypsum nucleation induction times. The gypsum precipitate quickly coated the calcite surface, forming arrow-like forms parallel to the crystallographic orientations of the calcite etch pits. Gypsum precipitation coupled with dolomite dissolution was slower than that of calcite, indicating the dissolution rate to be the rate-controlling step. The resulting gypsum coating partially covered the surface during the experimental duration of a few hours.

## Introduction

The overall process of dissolution of carbonate minerals and precipitation of gypsum is relevant in environmental settings, such as the treatment of acid mine drainage (AMD), geological CO_2_ sequestration and monument preservation. The use of limestone (calcite) in the treatment of AMD with elevated concentrations of heavy metals and sulfate is common [1–5]. The purpose is to retain metals and neutralize acidity by means of the so-called anoxic limestone drain (ALD) [1–5]. AMD, flowing through benches filled with calcite gravel, dissolves limestone and thereby increases the Ca^2+^ concentration, alkalinity and pH. Because, in general, AMD contains high concentrations of sulfate and metal ions, the dissolution of calcite initiates a coupled reaction chain that allows the system to precipitate sulfate as gypsum and metals (Al^3+^ and Fe^3+^) as hydroxides:

[1]
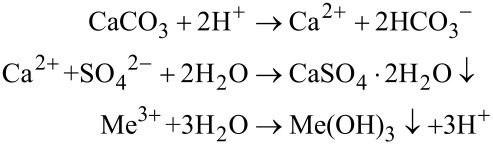


Such coupled processes, in which the dissolution of one phase produces a supersaturation of another phase in the fluid at the mineral-fluid interface and the new phase can precipitate, are well-documented [6–8].

AMD treatment becomes ineffective as soon as the precipitated hydroxides and/or gypsum fully coat the limestone surface and impede further dissolution of calcite. This mechanism is known as passivation or armoring [3,9–16]. While metal phases tend to precipitate between the calcite grains, gypsum tends to precipitate strongly adhered on the dissolving calcite surface, which is the main mechanism responsible for surface passivation [14–17]. This strong attachment of gypsum to the calcite surface results from crystallographic continuity between the two phases, namely “lattice matching” as pointed out by Booth et al. [18]. The fact that the crystallographic structure of gypsum and calcite exhibits parallel rows of cations and anions, and the cation–cation spacing for both minerals is 4.99 Å suggests a favourable overgrowth of the gypsum (010) plane on top of a calcite cleavage surface.

In the context of geological CO_2_ sequestration, the interaction between the acidic sulfate-rich brines and carbonate minerals of the reservoir rock promotes calcite dissolution and gypsum precipitation [18–22]. The effect of acid rain on historical monuments, buildings and statue degradation results from the dissolution of limestone by rain containing dissolved atmospheric SO_2_ and the subsequent precipitation of gypsum [23–25]. Large amounts of synthetic gypsum can precipitate during industrial processes involving the reaction between calcite and sulfuric acid [26].

The motivation of this study is to learn about the overall process of calcium carbonate mineral (calcite and dolomite) dissolution and gypsum precipitation in acid sulfate solutions at the micro–nanoscale by means of in-situ atomic force microscopy (AFM) experiments. This approach allows for a visualization of the processes occurring at the reacting carbonate surface.

In the literature, many studies deal with carbonate mineral reactivity [27–37]. In particular, the study of calcite dissolution and gypsum precipitation by Booth et al. [18] is relevant for our experimental AFM study as the authors provided SEM and AFM observations (in situ and ex situ) of the overall process of gypsum coating on calcite (causing passivation or armoring) at pH 1 and 2 in mixed HCl and Li_2_SO_4_ solutions. They reported on i) the reduction of calcite reactivity due to the gypsum coating, ii) the shape of gypsum crystals (rows parallel to the flux) and iii) the relation between anions and cations of the lattices of both calcite and gypsum. It is suggested that the likely match between cations favors the epitaxial overgrowth of the gypsum (010) face on top of the calcite cleavage plane.

In this study we attempt to enhance the current knowledge about the complementary processes of calcite/dolomite dissolution and gypsum precipitation. Two types of solution were used: (1) acid sulfate solution (Na_2_SO_4_) undersaturated with respect to gypsum and (2) acid sulfate solution (CaSO_4_) equilibrated with respect to gypsum. The experimental pH ranged from approximately 2 to 6 and the in-situ AFM experiments were run at ambient temperature (23 ± 1°C) and pressure.

## Experimental

The experiments were carried out by using a Digital Instruments (Bruker) Nanoscope III AFM equipped with a fluid cell sealed with an O-ring (50 μL volume), in contact mode using Si_3_N_4_ tips (Bruker, NP-S20) at room temperature (23 ± 1 °C). The scanning frequency was about 3 Hz and the image resolution was of 256 lines per scan, giving an average scan time of one image about every 100 seconds. The scan size ranged from 1 × 1 µm^2^ to 15 × 15 µm^2^. Images were analyzed with WSxM free software [38].

Single fragments of calcite (Iceland Spar, Chihuahua, Mexico) and crystalline dolomite (Eugui, Navarra, Spain) of approximately 4 × 3 × 1 mm (crystal volume ≈ 12 mm^3^) were cleaved immediately prior to experiments and attached to a fixed and oriented Teflon holder with commercial conductive carbon cement (CCC) and mounted in the fluid cell. The cleavage surface of calcite and dolomite is the (104) surface.

Acid solutions were prepared immediately before the experiments by adding the appropriate amounts of reactive analytical grade, CaSO_4_·2H_2_O (Merck pro analysis) and Na_2_SO_4_ (Grüssing purity 98%), to Millipore MQ water (resistivity = 18 MΩ·cm) (Table 1). The solution pH was adjusted to the chosen pH (approximately from 2 to 6) by adding concentrated H_2_SO_4_. Measurements of the pH were carried out by using a InoLab pH meter, equipped with a WTW Sentix 21 electrode calibrated with an accuracy of ±0.02 pH units. The electrode was calibrated with Crison buffer solutions at pH 4 and 7. The saturation index (SI) with respect to gypsum and calcite of the input solutions was calculated by using the PhreeqC code and the PhreeqC database [39].

**Table 1:** Experimental conditions.

experiment	substrate	pH	electrolyte	Ca_inp_ [mol/L]	Na_inp_ [mol/L]	SO_4inp_ [mol/L]	SI calcite	SI gypsum

cal14	calcite	2.23	Na_2_SO_4_	—	5.42E−02	3.10E−02	—	—
cal12	calcite	2.20	Na_2_SO_4_	—	4.62E−02	2.70E−02	—	—
cal9	calcite	2.18	CaSO_4_	1.60E−02	—	2.50E−02	−11.0	0.05
cal10	calcite	2.18	CaSO_4_	1.60E−02	—	2.50E−02	−11.0	0.05
dol6	dolomite	2.11	Na_2_SO_4_	—	1.02E−02	1.00E−02	—	—
dol3	dolomite	2.11	Na_2_SO_4_	—	2.62E−02	1.80E−02	—	—
dol4	dolomite	2.18	CaSO_4_	1.60E−02	—	2.50E−02	−11.0	0.05
dol1	dolomite	2.14	Na_2_SO_4_	—	5.02E−02	3.00E−02	—	—

cal19	calcite	3.37	Na_2_SO_4_	—	5.56E−02	2.70E−02	—	—
cal8	calcite	3.06	CaSO_4_	1.50E−02	—	1.60E−02	−9.2	0.00
cal21	calcite	2.92	Na_2_SO_4_	—	1.12E−02	6.00E−03	—	—
dol5	dolomite	3.00	CaSO_4_	1.50E−02	—	1.60E−02	−9.2	0.00
dol7	dolomite	3.00	Na_2_SO_4_	—	2.70E−02	1.40E−02	—	—

cal4	calcite	4.08	CaSO_4_	1.50E−02	—	1.50E−02	−7.1	−0.01
cal2	calcite	4.03	Na_2_SO_4_	—	1.12E−02	6.00E−03	—	—

cal6	calcite	4.80	CaSO_4_	1.50E−02	—	1.50E−02	−5.7	−0.02

cal3	calcite	5.82	CaSO_4_	1.50E−02	—	1.50E−02	−3.7	−0.02

**Figure 1:**
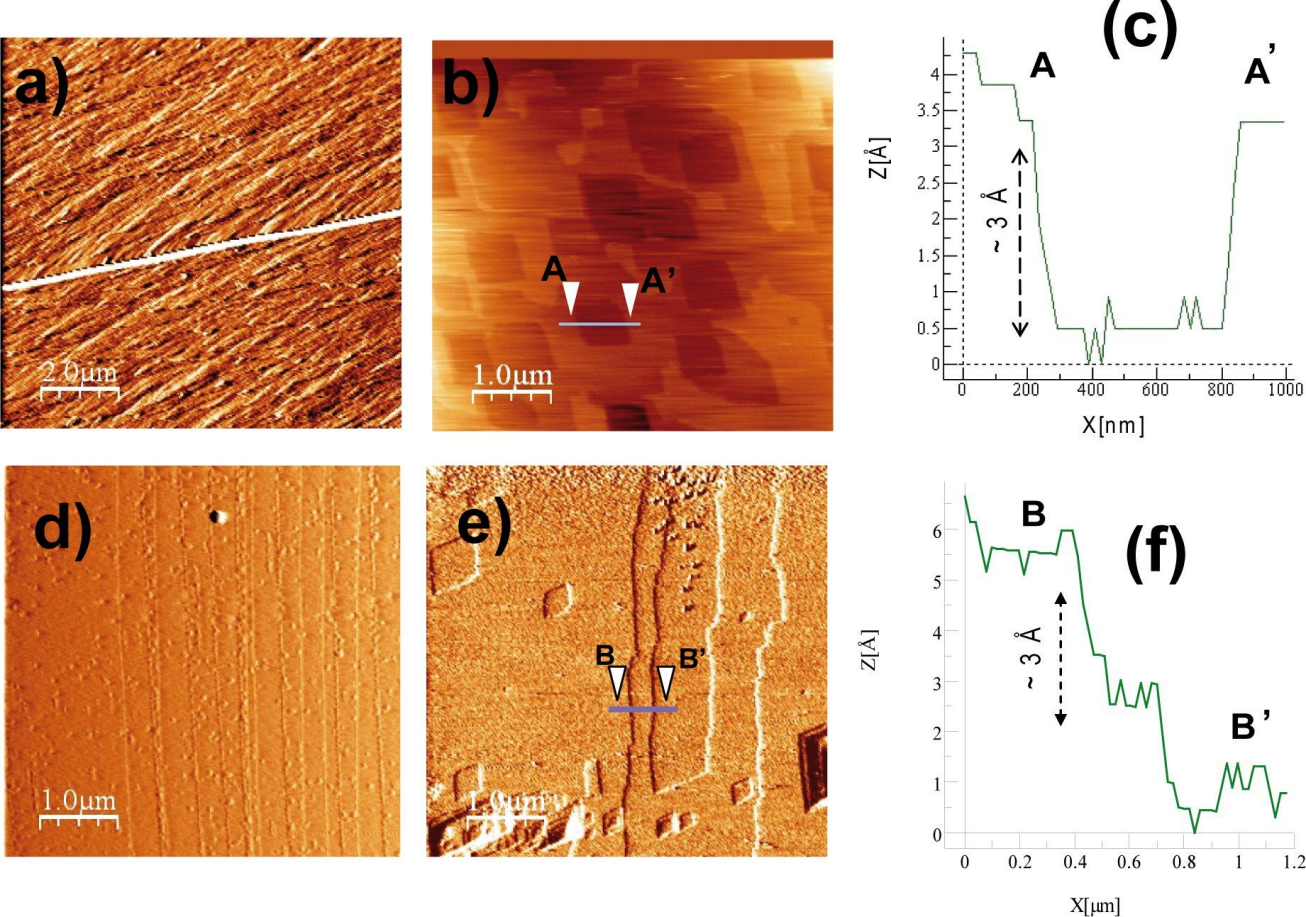
AFM deflection images of calcite cleavage surfaces. Top row: a) image in air shows the initial flat surface with a topographic variation that ranges over 2 nm. The white line across the image corresponds to a terrace; b) same surface region with some drift after 300 s in Millipore MQ water showing a high density of etch pits homogeneously distributed and c) depth profile of an etch pit section. Bottom row: d) image in air shows the initial flat surface with a topographic variation that ranges over 4 nm and e) same surface region after 240 s in Millipore MQ water showing the random formation of etch pits and f) depth profile of a step edge section shown by the arrows in e).

The experimental strategy consisted of three stages. First, prior to each in-situ experiment an in-air image of a selected region of the cleaved surface was taken to examine the initial topography and surface features of interest (flat/rough areas, steps terraces and edges; Figure 1a and Figure 1d). Secondly, after an appropriate region of the cleavage surface was selected, the Millipore MQ water was injected by using a syringe to fill the available volume of the fluid cell containing the sample (ca. 38 μL) and flow over the mineral surface. Renovation of the Millipore MQ water was performed after each sequential image capture (ca. 1.5 min) to ensure a similar bulk solution concentration as the reaction took place during the experiment and prevent a saturation of the solution during the reaction (close-to-equilibrium approach). During this stage the calcite dissolution rate, *R*_AFM_ (mol·cm^−2^·s^−1^), was obtained from the dissolved volume of calcite created by the etch pits (as described by Urosevic et al. [37]):

[2]
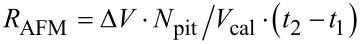


[3]
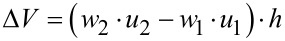


where Δ*V* is the increase in dissolved volume of an etch pit between *t*_2_ and *t*_1_ in two sequential images, *w*, *u* and *h* are the width, length and depth, respectively, of an etch pit (*h* remains constant at ca. 0.3 nm), *N*_pit_ is the average number of etch pits per cm^2^, and *V*_cal_ is the molar volume of calcite (31.20 cm^3^·mol^−1^). By using sequential images, the pit expansion rate, *R*_s_ (nm·s^−1^), was also calculated from the variation in length of the etch pit sides (Δ*w* or Δ*u*) over time (*R*_s_ = Δ*w*/(*t*_2_ − *t*_1_)). Likewise, the step velocity, *R*_T_ (nm·s^−1^), was calculated from the increase in terrace width (Δ*L*) over time (*R*_T_ = Δ*L*/(*t*_2_ − *t*_1_)). After the conclusion of mineral dissolution in Millipore MQ water, the third stage started as the cell was filled with the chosen sulfate-rich acid solution in order to promote the precipitation of gypsum. During this stage, solution renovation was not allowed. Hence, the solution saturation state approached an equilibrium with respect to the dissolving carbonate mineral.

Micro-Raman analysis was used to identify the newly precipitated sulfate phases on the calcite and dolomite cleavage surfaces. Micro-Raman spectra were obtained by using a dispersive spectrophotometer Jobin-Yvon LabRam HR 800 with 532 nm light for sample excitation and a CCD detector cooled to −70 °C. The laser power used was between 0.5 and 4 mW. The spectrophotometer was coupled to an optical microscope Olympus BXFM with 50× and 100× objectives. The samples were dried before measurement.

## Results and Discussion

### Dissolution of calcite

**Figure 2:**
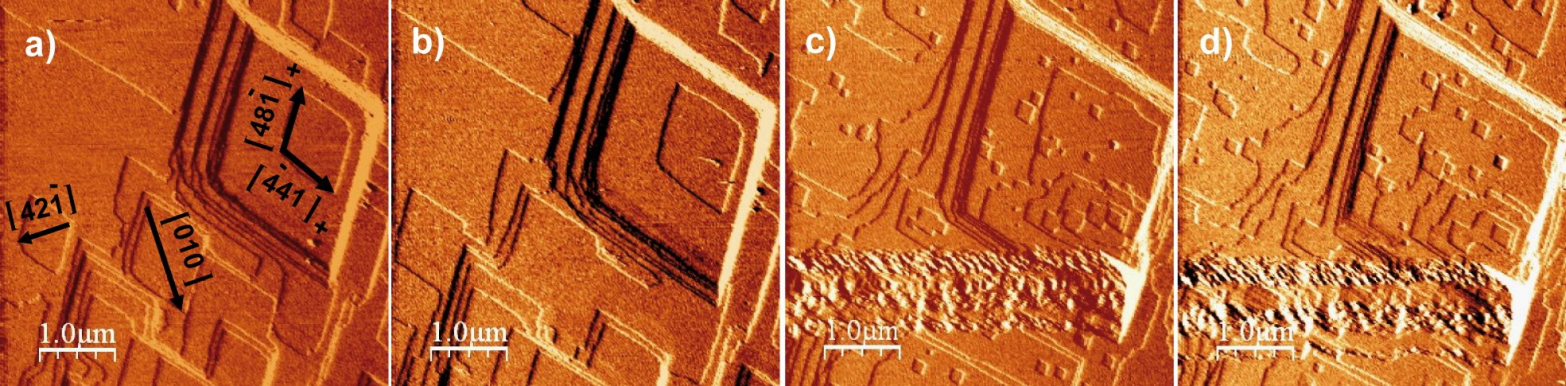
Sequential AFM deflection images of the reacted calcite (104) surface: initially in Millipore MQ water (a and b) and acid solution (pH 4.80) (c and d). Etch pits developed and spread. As pH was decreased to 4.80, a large population of etch pits suddenly formed. Rhombohedra formed along the 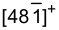 and 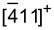 directions with the long and short diagonals parallel to [010] and 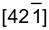, respectively.

**Figure 3:**
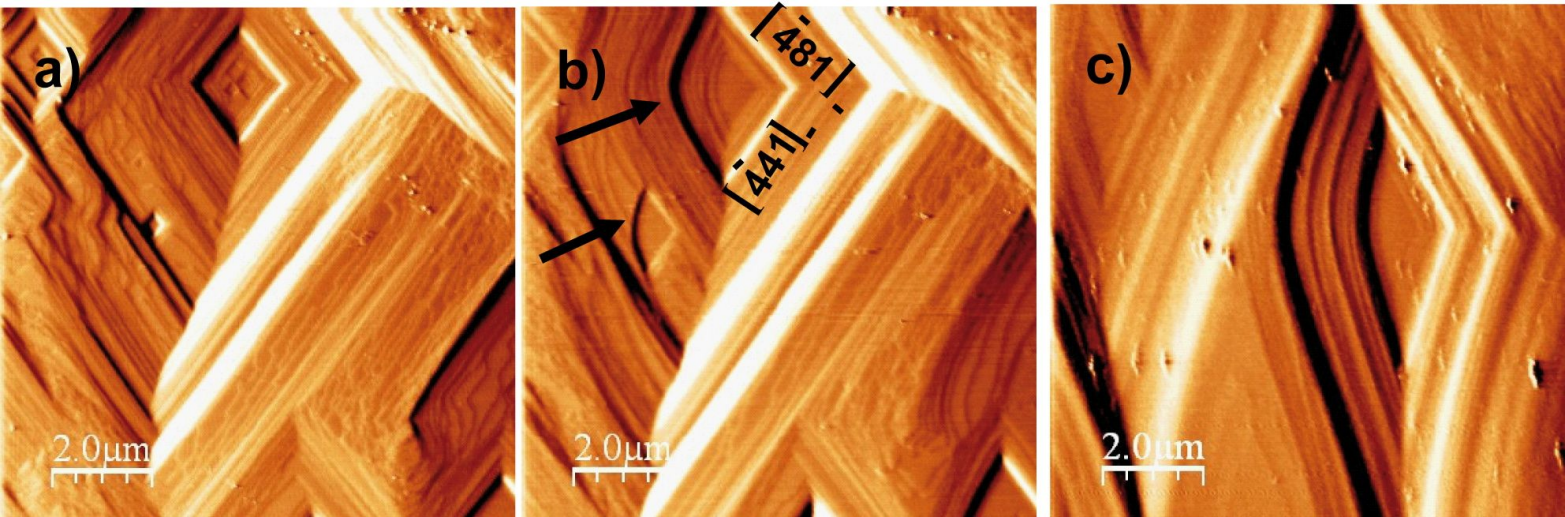
Sequential AFM deflection images of the reacted calcite cleavage surface in contact with Na_2_SO_4_ solution: a) characteristic morphology of rhombohedral etch pits (after acid injection, pH 4.08) and b) rounding of the obtuse–obtuse corner of the rhombohedral etch pits (shown by arrows) after 12 min, and c) rhombohedral etch pit with elongated shape after 43 min with a short/long diagonal ratio of 0.35 ± 0.02.

**Figure 4:**
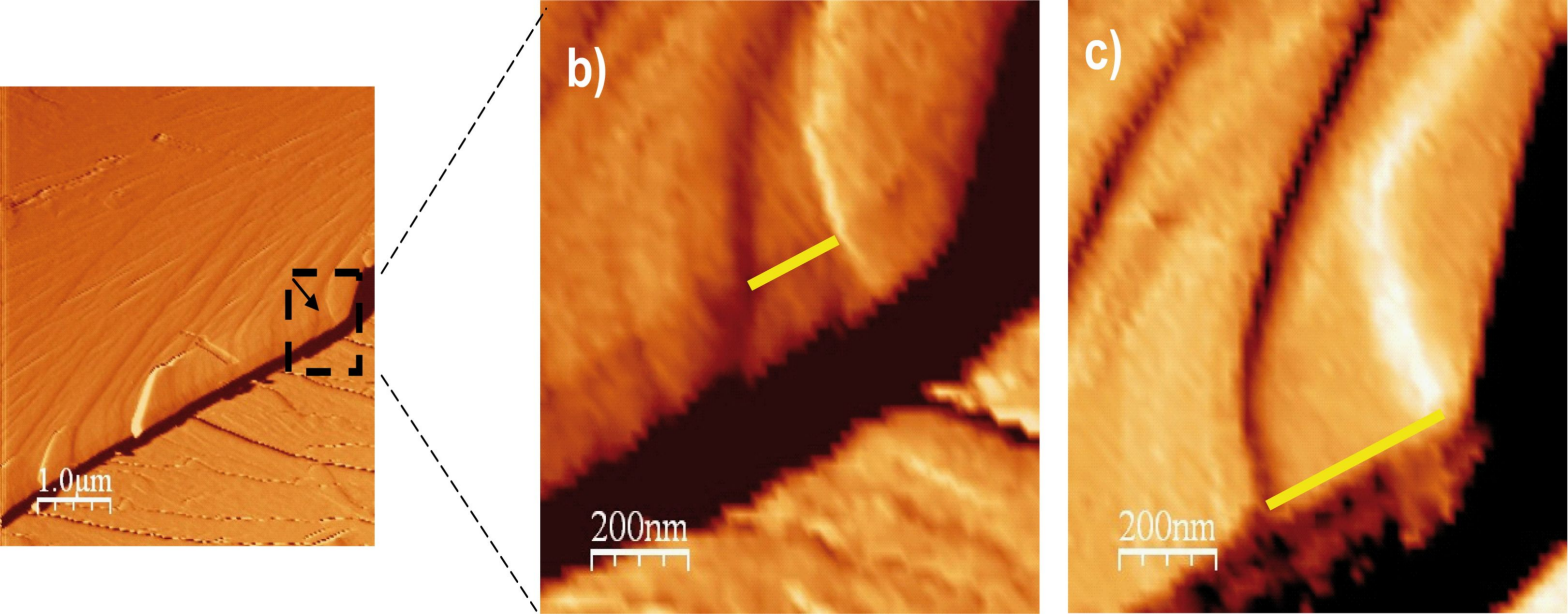
AFM deflection images of dolomite dissolution in Millipore MQ water: a) in air image of the dolomite (010) surface (exp. dol 1 in Table 1). Selected squared region in (a) to calculate the step-retreat rate based on the variation in length with time of the pointed terrace. The sequential images in b) and c) after 7.5 and 11.5 min respectively, show the consequent terrace evolution.

**Figure 5:**
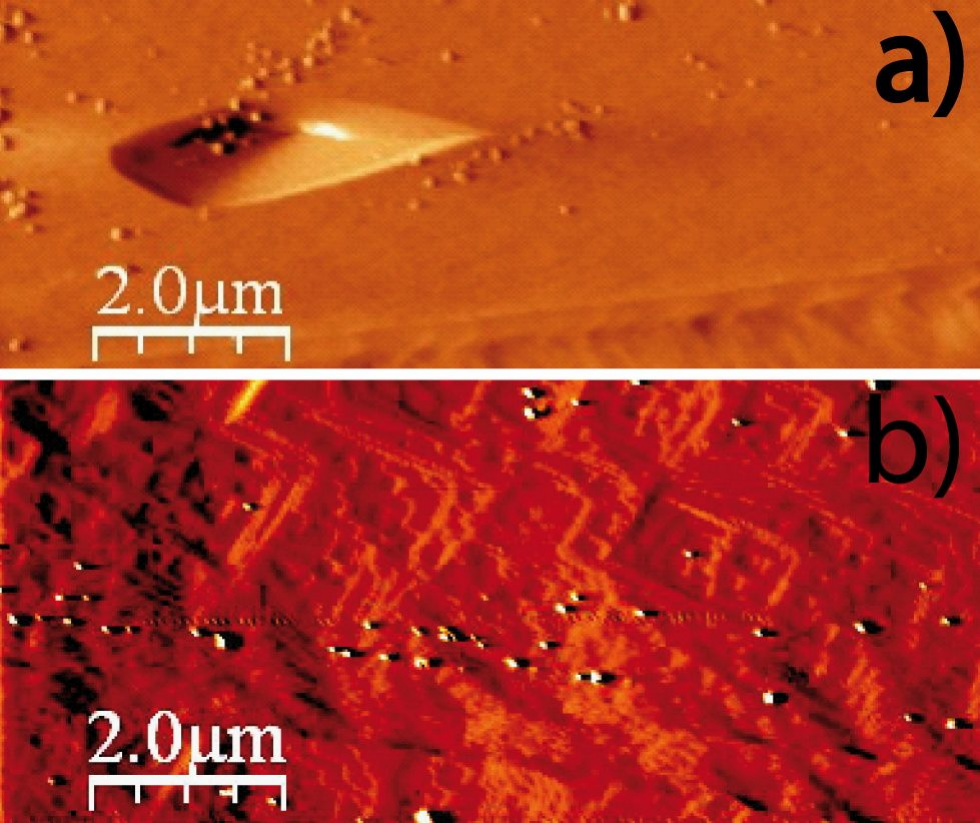
AFM deflection images of the reacted dolomite (104) cleavage surface in acid Na_2_SO_4_ solutions: a) after 10 min in pH 2, isolated etch pits were observed and b) in pH 3, nucleation of etch pits was observed all over the surface after 8 h.

Dissolution of the (104) calcite surface in Millipore MQ water was readily observed. Figure 1b and Figure 1c show the formation of shallow (depth ≈ 0.3 nm ≈ calcite unit cell) and deep rhombohedral etch pits all over the surface [19,30,36,40]. The ratio between the etch pit rhombus diagonals was 0.71 ± 0.02, which is similar to that reported by Pérez-Garrido et al. [41]. Etch pit merging and formation of trenches or steps were observed (Figure 1b and Figure 1e). The number of etch pits per square centimeter of surface (*N*_pit_) varied from 8 × 10^7^ (only etch pits, Figure 1b) to 5 × 10^8^ (etch pits and steps, Figure 1e) in scanned flat regions with similar initial roughness. The measured calcite dissolution rate, *R*_AFM_, was 1.45 × 10^−10^ mol·cm^−2^·s^−1^, which agrees with that at nearly neutral pH reported elsewhere [19,42–43]. The etch pit expansion rate, *R*_s_, was measured to be 1.82 ± 0.12 nm·s^−1^ and falls within the range of those calculated for deionized water by Jordan and Rammensee (velocity of slow step 0.5 ± 0.2 nm/s and of fast steps 2.5 ± 0.5 nm/s) [44].

Interaction between the acidic sulfate-rich solutions and the calcite cleavage surface (solution injected and not renewed) induced faster dissolution than in Millipore MQ water. A massive nucleation of new rhombohedral etch pits took place at pH 4.80 after solution injection, in contrast to the fairly regular distribution of etch pits in Millipore MQ water (Figure 2). At pH 4.80 *R*_AFM_ was 5.50 × 10^−10^ mol·cm^−2^·s^−1^, which is faster than that at pH 7, and agrees with the expected rate at pH 5 and 25 °C [19].

In the experiments with Na_2_SO_4_ solution (Figure 3a; solution injected and not renewed) the dissolution of the calcite cleavage surface was taking place such that equilibrium with respect to calcite was being approached. It was observed that the shape of newly formed rhombohedral etch pits was changing with time as the solution approached equilibrium with respect to calcite. The evolving shape was characterized by rounding of the obtuse–obtuse corner (Figure 3b–d). According to Teng et al. [45] and Teng [46] the retreat velocities of acute and obtuse steps do not show a linear dependence on supersaturation. In addition, several studies have shown that the velocities of acute and obtuse step spreading have different sensitivities to the solute activity ratios in the solution [32,36,47]. Calcite dissolution continuously took place during the solution saturation state drift. This implies a change in Gibbs energy along the experimental runs. As pointed out by Stipps et al. and de Leeuw et al. [48–49] the observed distortion of the etch pit shape (Figure 3b and Figure 3c) likely corresponds to an increase in the difference of velocities between obtuse and acute steps.

### Dissolution of dolomite

Dolomite dissolution experiments were carried out similarly to those of calcite. First, dolomite dissolved in Millipore MQ water, and then, the reaction took place in sulfate-rich solutions at pH 2 and 3 (Table 1). Contrary to calcite dissolution, when dolomite reacted in Millipore MQ water, a nucleation of etch pits was not observed for approximately 25 min. Only, at specific surface localities, step retreat was observed (Figure 4a), allowing the calculation of the retreat velocity *R*_S,_ considered to be the average retreat velocity of non-crystallographically equivalent steps (Figure 4b and Figure 4c), which was 0.14 ± 0.03 nm·s^−1^. This value is not far from the etch spreading rate of 0.09 ± 0.01 nm·s^−1^ reported by Urosevic et al. [37] and is about one order of magnitude lower than the etch pit expansion rate of calcite obtained in this study.

As dolomite reacted in acid solution, etch pit nucleation of isolated etch pits was observed over the cleavage surface after 10 min. Single etch pits presented an elongated rhombohedral shape (Figure 5a). As the surface kept dissolving for 8 h, etch pit nucleation occurred all over the surface. Lack of sequential images for this long run prevented us from calculating *R*_AFM_ under acid conditions (Figure 5b). The formed etch pits showed the typical rhombohedral shape as expect from carbonate mineral dissolution [37].

### Coupled dissolution of calcite and dolomite and precipitation of gypsum

**Figure 6:**
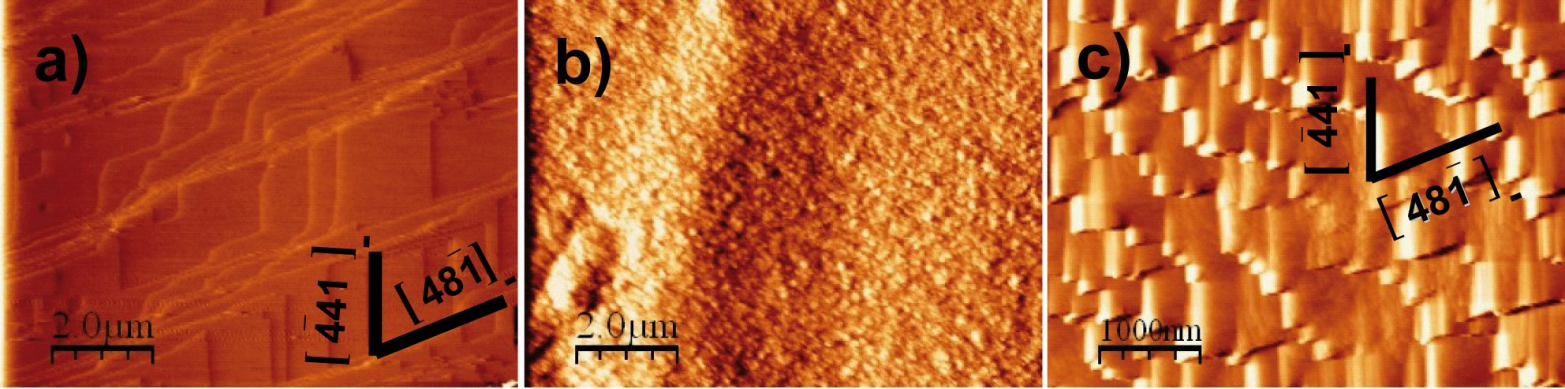
AFM deflection images of reacting (104) calcite surface: a) dissolution in Millipore MQ water; b) after injecting a solution in equilibrium with gypsum at pH 2.18, gypsum precipitation starts (1.5 min) and c) gypsum arrows grow laterally and coalesce (41 min).

As the calcite (104) cleavage surface reacted with the pH 2 solution equilibrated with respect to gypsum, gypsum precipitation was readily observed (Figure 6). Micro-Raman analyses of the retrieved reacted samples confirmed the presence of gypsum. Gypsum nucleation took place uniformly all over the calcite surface immediately after the acid solution interacted with the dissolving cleavage surface (Figure 6a and Figure 6b). At pH 2, the gypsum precipitation induction time was slower than 100 s (time between two sequential image captures). The epitaxially grown gypsum crystals displayed an elongated (arrow-like) shape, consistent with their crystallographic monoclinic form, usually presented as tabular crystals, with the long and short sides parallel to the calcite 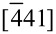 and 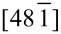 directions, respectively (Figure 6a and Figure 6c).

This crystal morphology was observed by Booth et al. [18]. 3-D images of the arrow-shaped gypsum crystals showed that the formed gypsum crystals, which entirely coated the cleavage surface, were slightly tilted (ca. 1°) with respect to the calcite (104) cleavage surface. The lack of a reference surface on the calcite substrate and the fast-formed gypsum coating prevented the calculation of gypsum growth rates at the pH range studied. Gypsum precipitation ceased as Ca release from calcite dissolution stopped. This was most likely because calcite dissolution stopped as either the entire calcite surface was totally passivated impeding ion release through the gypsum layer, or because equilibrium with respect to calcite was achieved.

**Figure 7:**
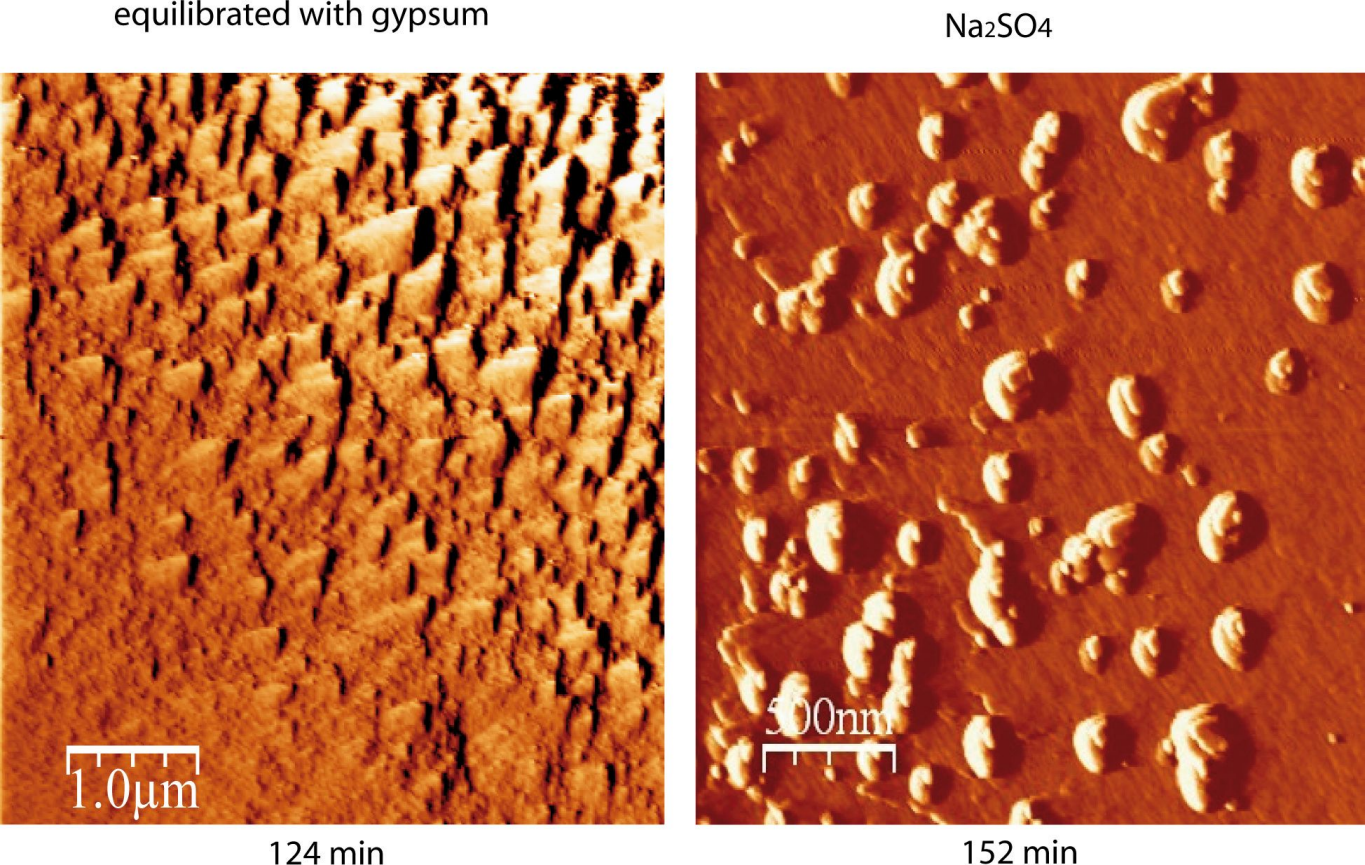
Gypsum precipitation on a calcite surface at pH 3: a) Experiment with gypsum equilibrated CaSO_4_ solution: homogeneous, arrow-type gypsum growth on the cleavage calcite surface; b) Experiment with Na_2_SO_4_ solution: random protuberances over the calcite surface.

In experiments in which calcite dissolved at pH ≥ 3 in gypsum equilibrated solutions, the gypsum induction time was longer than 240 s, indicating slower gypsum growth than that at pH 2 due to slower calcite dissolution. Gypsum also grew epitaxially over the entire surface and, in general, the crystals showed the arrow-like shape (Figure 7a). In some Na_2_SO_4_ experiments, however, gypsum precipitation occurred non-uniformly over the cleavage surface, taking place at specific localities, mostly at step edges, and forming individual protuberances (spikes), suggesting preferential sites for the formation of these nuclei (Figure 7b).

**Figure 8:**
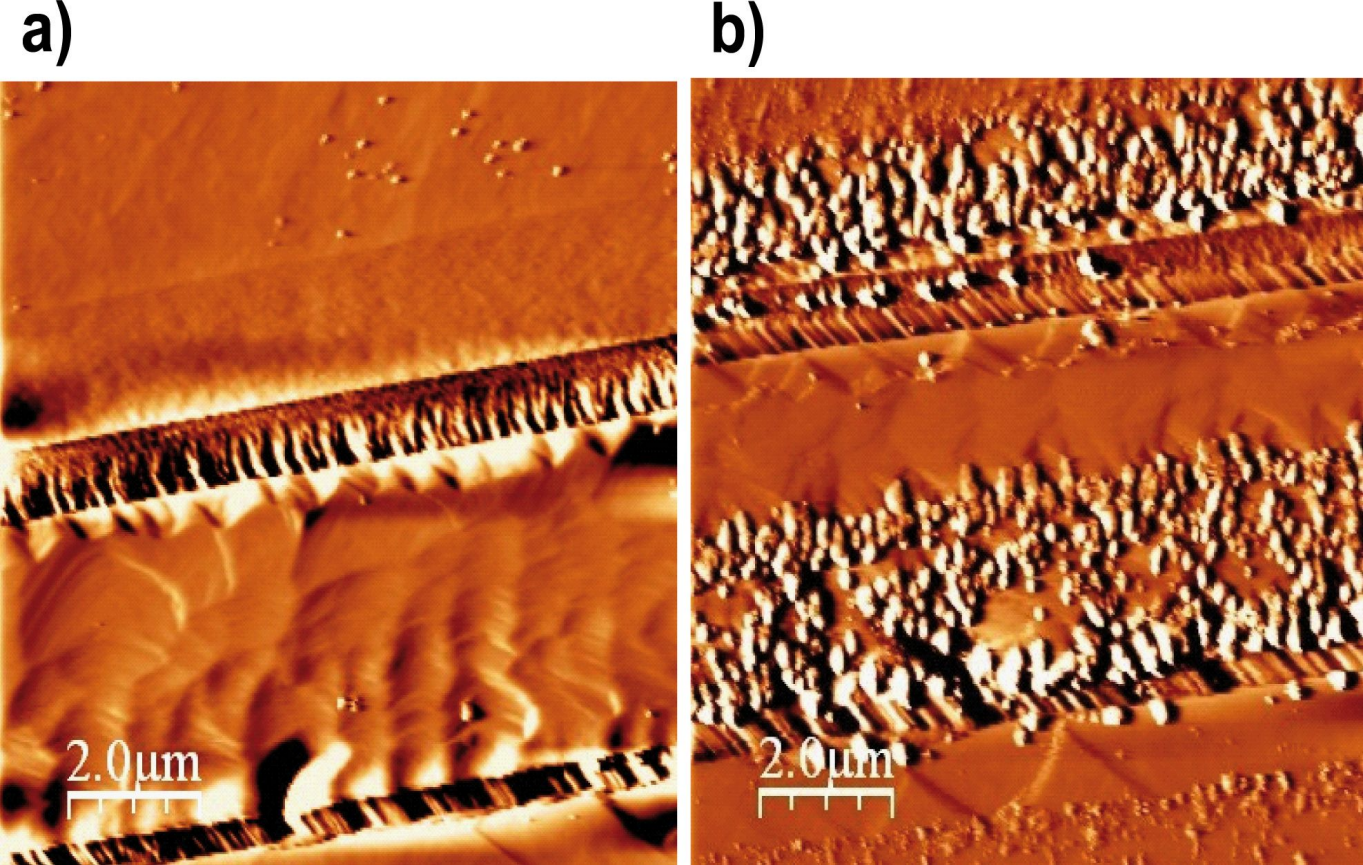
Sequential AFM deflection images of reacted dolomite surface in pH 3 (H_2_SO_4_) in solution equilibrated with respect to gypsum: a) after 4 h, shallow and deep etch pits are visible on the dolomite surface and b) after 6 h, gypsum precipitated mainly along the step edges.

When the cleaved dolomite surface was the substrate, gypsum precipitation from dolomite dissolution was slower than that from calcite dissolution at the same pH. Micro-Raman analyses of the reacted fragments at pH 2 and 3 confirmed precipitated gypsum at the dolomite cleavage surfaces. Gypsum precipitation occurred on the previously etch pitted dolomite surface after about 6 h, and again it was difficult to establish an induction time. Epitaxial growth was observed to be non-uniform over the surface (Figure 8), taking place on preferential surface regions, such as step and terrace edges, and areas with marked roughness. This behavior suggests that gypsum precipitation on dolomite cleavage surfaces was favored at highly reactive surface regions, where dolomite dissolution and hence element release was highest. After 8 h of reaction time, dolomite passivation was still only partial with etch pitted regions still visible, in contrast to the full gypsum armoring on the calcite surface.

## Conclusion

In-situ atomic force microscopy was used to investigate the coupled processes of carbonate mineral dissolution and gypsum precipitation in acid sulfate-rich solutions in solutions both undersaturated and in equilibrium with respect to gypsum at room temperature.

Dissolution of calcite and dolomite occurred forming the characteristic rhombohedral etch pits. Calcite dissolution rates measured at nearly neutral pH and pH of 4.80 agreed with VSI-measured rates [19]. The calcite etch pit expansion rate and the dolomite step retreat velocity were calculated in near neutral pH (Millipore MQ water), the latter being about one order of magnitude lower than the former. Precipitation occurred as a result of the carbonate mineral dissolution. Therefore, as in acidic pH conditions calcite dissolution rates were faster than those of dolomite, gypsum precipitation was correspondingly faster in the calcite dissolution experiments. Epitaxial growth was the growth mechanism as observed by Booth et al. [18], and gypsum nucleation induction times were shorter in the calcite dissolution experiments. In the case of calcite dissolution in gypsum-equilibrated solutions, gypsum nucleation occurred immediately and surface coating was uniform all over the calcite surface, yielding a total calcite passivation. Arrow-shaped gypsum crystals evolved along the etch pit crystallographic directions (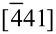 and 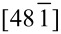). In Na_2_SO_4_ solutions undersaturated with respect to gypsum, precipitation occurred via the formation of isolated growth protuberances randomly distributed over the cleavage surface. In the case of dolomite dissolution in gypsum-equilibrated solutions, gypsum precipitation was favored at highly reactive surface regions (step and terrace edges) and rough regions. Gypsum partially coated the dolomite surface during the experimental runs.

In all experiments gypsum precipitation resulted from a two-step process: 1. The calcite or dolomite dissolved, as observed in the regular formation of rhombohedral etch pits and step retreat, thereby releasing Ca^2+^ or Ca^2+^ and Mg^2+^ ions to solution. 2. The solution at the mineral–solution interface became supersaturated with respect to gypsum, which then precipitated. These two processes were coupled at the interface and continued as long as Ca^2+^ was being released.
